# The Sole Test of Sanity

**Published:** 1918-09-14

**Authors:** 


### PAPERS ON MADNESS.

### IV.—The Sole Test of Sanity.

In the previous papers we have given a brief summary of the history of the treatment of mad people in the last century, and of the gradual changes in the law with respect to their personal care. This is an aspect of madness that cannot be disregarded, and is, indeed, of paramount importance, for, unlike medical and surgical diseases, madness has of necessity a social and legal bearing. Other diseases impair the patient's capacity of action. They render him less capable of acting in this or that manner. If a man breaks his leg, he is less capable of walking. If he has a whitlow on his finger, he is less capable of manipulation. If he has valvular disease of the heart, he is less capable of strenuous exertion. If he has fever, he is less capable of exertion of any kind. But medical and surgical diseases, though they impair the quantity of action of which a man is capable, do not impair its quality. They render action less efficient, and when severe they may almost abolish the patient's capacity of acting; but he always retains some capacity of acting, even if it is only that of speaking; and whatever impairment of action there may be is impairment of quantity only, and does not affect the quality of his action except in so far as less efficient action is of necessity substituted for the more efficient action of which the healthy man is capable. In other words, as the healthy man has the capability of changing his mode of action to suit changing circumstances, so the man suffering from medical or surgical disease has the capability of changing his action to suit the change in his bodily condition. Action is the adaptation of one's self to one's circumstances; and needs modification according as the circumstances change, and according as the self changes; and as long as we retain the capacity of altering our action so as to suit any change that may occur either in our circumstances or in ourselves, so long we retain our sanity.

### What is Sanity?

Madness is the loss of this power of adapting our action to suit our circumstances. If a man's circumstances change in such a manner as to affect his welfare, he will, as long as he is sane, alter his action so as to adapt himself to the change. If the weather becomes cold, he will light a fire, or put on more clothing, or both; and *vice versâ* if the weather turns hot. If his income increases or diminishes, he will increase or diminish his expenditure accordingly. If a new law that affects him is passed, he will alter his conduct so as to conform to it. As his children arrive at an educable age, he will take measures for their education. He will change his action according to his changing circumstances, and as long as his circumstances do not change he will not change his settled action towards them unless he himself is changed. But any change in himself must be met by change in his action towards his circumstances, or the adaptation of self to circumstances in which sanity consists will fail. When he is a child, he acts as a child. His ways are boisterous, and display his overflowing energy. He plays marbles, or spins his top, or trundles his hoop, and frequents the tuck-shop, and finds delight in doing so; but when he becomes a man, he puts away childish things. A man does not play marbles, or spin tops, or trundle hoops, or frequent the tuck-shop, and if he did we should regard him as mad. Why? Manifestly because such action no longer effects that adjustment, in which normal action consists, of self to circumstances.

### Adjustment between Self and Circumstances.

Normal action is such as to adjust the relation between the self and the circumstances, either by altering the circumstances, as when we put on more clothes in cold weather; or by altering ourselves, as when we learn a new language on going to a new country; or by altering our action, as when we stop at home and go to bed instead of going to business when we find ourselves suffering from fever. The relation between the self and the circumstances is thrown out of adjustment whenever there is a change in the self or a change in the circumstances, or a change in the relation of the one to the other, and every such change in the relation must be met by a readjustment, which is effected either by changing the self, or by changing the circumstances, or by changing the relation of the one to the other; and as long as the adjustment is complete, and no change takes place in either of the three conditions, action must be steadily maintained unchanged, or the adjustment will be disturbed and thrown out, and readjustment will be required. Action which brings about or maintains the due adjustment of the relation between self and circumstance is sane action, and sanity consists in action of this kind. Action which disturbs the relation between self and circumstances and throws them out of adjustment is erroneous action, and may be merely sane mistake or may be mad action.

### The Madman's Persistence.

There is, therefore, a certain similarity between error and madness. All mad action is erroneous, but erroneous action is not necessarily mad, and it is very important to find the true distinction between them, for in practice they are often confused. The distinction is this: a sane person who does a mistaken act recognises, by the failure of the act to achieve his purpose, that the action is mistaken, and alters his mode of action accordingly. At the least he refrains from pursuing that mode of action as soon as it appears manifest that it will not achieve his purpose. The madman who does a mad act does that which is mistaken—that which will not achieve his ultimate purpose, that which fails to adjust or readjust himself to his circum-

☛ Previous articles appeared on March 2, August 24 and 31, page 469.

**Papers on Madness**—(*continued*).

stances or his circumstances to himself and his requirements. But when his action fails to achieve his purpose he does not change it. He persists in it. It may be, and often is, that his action is of such a nature that it could not achieve any useful or desirable purpose, but he does it nevertheless. He does not recognise the uselessness or the actual hurtfulness of his act, and he does that which is manifestly, and on the face of it, useless, undesirable, or harmful to himself and his interests; and persists in the action even when its uselessness, undesirability, or harmfulness is demonstrated by actual experience. This is the important difference between sane mistake and madness. The one can be corrected by the actor, the other cannot; and by observing whether the action is, on the face of it, useless, undesirable, or harmful, or whether, if not so on the face of it, it is persisted in even after its ultimate uselessness, undesirability, or harmfulness is become plainly apparent, we may judge without fail whether the action is sane or mad.

It will be seen from this description that the test of sanity or madness is the same as, according to Demosthenes, is the test of good oratory. It is action, and again action, and yet again action. It is by action, it is by behaviour, it is by conduct, that we judge whether a person is mad or not. This is the test, and this is the sole test. By no other means can we solve the problem.

### The Teaching of the Benighted.

For many generations it was held and taught that madness is disorder of mind, and it is still so held and taught by a few belated and benighted teachers in London, who know no better, and cannot assimilate new ideas. London doctors have always been conservative to the point of obscurantism with respect to all ideas that were not originated among themselves. This is well illustrated by the reception they gave to Listerism, as described in these pages, and it is illustrated again in the reception they have accorded to the new doctrine of madness. The younger men have adopted it. It is adopted more or less generally in the provinces, and it is widely adopted in Scotland, for the Scotch are but little hidebound by tradition; but the worthy old fogies who lead the profession of alienism in London lead it as Mr. Asquith led the nation, by standing still, and waiting to see which way the cat is going to jump. Now that the animal has taken its leap, no doubt those who are not too old to change will follow it.

Of course, there is always disorder of mind in madness, just as there is always pain—which also, by the way, is disorder of mind—in cancer; but pain is not the same thing as cancer, and cancer does not consist of pain alone; and similarly, though disorder of mind is always present in madness, disorder of mind is not the same thing as madness, and madness does not consist in disorder of mind alone. It is manifest to everyone but the pundits who practise in insanity that we cannot see or witness in any way what is passing in another person's mind. We can only judge of it to the best of our ability by what he does and says, and by his facial expression, bodily attitude, and, in short, the way in which he behaves; and as long as his conduct and behaviour are normal, it matters not in what condition his mind may be, or whether he has any mind at all; he is to all intents and purposes a sane man, and we should never dream of regarding him otherwise than as a sane man. Not even the alienists, who say that madness is disorder of mind, would consider him mad.

### Disorder of Conduct Enough.

On the other hand, when a man acts as a madman and behaves as a madman, when he tears up his clothes and smashes the furniture; when he walks into the street stark naked; when he squanders money with reckless disregard of his solvency; when, not being incapacitated by bodily illness, he sits or lies all day in motionless lethargy; when he is indifferent to the calls of Nature, or responds to them without regard to decency or comfort; when he outrages the conventions of civilised society; when he moans incessantly over misfortunes that have not happened, or rejoices extravagantly over the reception of wealth, titles, and honours that he has not received; then we regard him as mad, then we are ready to certify him as mad, and have no hesitation in certifying and treating him as a madman, without making any inquiry at all into the condition of his mind. The state of his mind is beside the question. We may know or guess that it is disordered, but we do not investigate the disorder or take any account of it. The disorder of conduct is enough. It is all we need consider, and all we do consider. He is certified as mad because he behaves as a madman, and for no other reason.

### The Mental Side of Giddiness.

Moreover, a man's mind may be disordered, and greatly disordered, without any suspicion of his madness being entertained for a moment. Setting aside such mental disorders as obsession and imperative idea, which, though they are often completely sane, are yet bordering upon madness, it is well known that hallucinations, that is to say, the perception or quasi-perception of sights and sounds that are imaginary, pretty often occur in persons whose sanity cannot be questioned, and never is questioned; and besides this, it is very insufficiently appreciated that giddiness has a mental side. It is a "feeling" or "sensation" of giddiness, and is so far mental, and of course disorder of mind. But is a man mad because he is giddy, or when he is giddy? A question not to be asked.

